# The prevalence and risk factors of cytomegalovirus infection in inflammatory bowel disease in Wuhan, Central China

**DOI:** 10.1186/1743-422X-10-43

**Published:** 2013-02-01

**Authors:** Fengming Yi, Jie Zhao, Rishi Vishal Luckheeram, Yuan Lei, Changgao Wang, Sha Huang, Lu Song, Wei Wang, Bing Xia

**Affiliations:** 1 Department of Gastroenterology, Zhongnan Hospital of Wuhan University School of Medicine, Donghu Road 169, Wuhan 430071, P.R. of China; 2 The Hubei Clinical Center & Key Laboratory of Intestinal & Colorectal Diseases, Wuhan 430071, P.R. of China

**Keywords:** Inflammatory bowel disease, Ulcerative colitis, Crohn’s disease, Cytomegalovirus, Risk factors

## Abstract

**Background:**

The etiology of inflammatory bowel disease (IBD) is not clear and cytomegalovirus (CMV) infection is often associated with IBD patients. The etiologic link between IBD and CMV infection needs to be studied. The objective of the present study is to investigate the prevalence and risk factors of CMV in a cohort of IBD patients from Central China.

**Methods:**

Two hundred and twenty six IBD patients (189 ulcerative colitis (UC) and 37 patients with Crohn’s disease (CD)), and 290 age and sex matched healthy controls were recruited. CMV DNA was detected by nested PCR, while serum anti-CMV IgG and anti-CMV IgM was determined by ELISAs. Colonoscopy/enteroscopy with biopsy of diseased tissues and subsequent H&E stain were then conducted in IBD patients with positive anti-CMV IgM. Finally, we analyzed the prevalence and clinical risk factors of CMV infection in IBD patients.

**Results:**

The prevalence of CMV DNA and anti-CMV IgG positive rate in IBD patients were 84.07% and 76.11%, respectively, higher than those in healthy controls (59.66% and 50.69%, respectively, *P* < 0.05), However, anti-CMV IgM positive rate was no different with healthy controls (1.77% vs 0.34%, *P* = 0.235). In univariate analysis of risk factors, the recent use of corticosteroid was associated with increase of CMV DNA and IgM positive rate in UC (P = 0.035 and P = 0.015, respectively), aminosalicylic acid drug therapy was correlated with positivity of CMV DNA and IgG in UC and CMV DNA in CD (P = 0.041, P < 0.001 and P = 0.014, respectively), the treatment of immunosuppresent was correlated with CMV IgM (P < 0.001). Furthermore, patients with severe UC were significantly associated with CMV DNA and IgM (P = 0.048 and P = 0.031, respectively). Malnutrition (albumin < 35 G/L) was also found to be related with CMV recent infection (P = 0.031). In multivariate analysis of risk factors in UC, pancolitis was significantly associated with CMV DNA positivity (*P* = 0.001). Severe UC and pancolitis seemed to be related with IgG positivity. For CD, there was just single factor associated with CMV positive in each group, multivariate analysis was unnecessary.

**Conclusions:**

CMV positive rate in IBD patients was significantly higher, than in healthy controls. The use of aminosalicylic acid, corticosteroid, immunosuppressants, pancolitis and severe IBD patients seemed to be more susceptible to CMV infection in univariate analysis of risk factors. However, no risk factor was found to be significantly correlated with CMV infection in multivariate analysis of risk factors.

## Introduction

Inflammatory bowel disease (IBD) including ulcerative colitis (UC) and Crohn’s disease (CD), consists of chronic, non-specific inflammatory diseases of the gut with unknown etiology. According to a retrospective survey, the incidence and prevalence of IBD in China are on the rise [1], and IBD is gradually become one of the refractory intestinal diseases. Even though the etiology of IBD is unknown, recent studies have shown that the pathogenesis of IBD is related to susceptible genes, immune dysregulation and microbiota of the gut.

Cytomegalovirus (CMV) is a β herpes virus with double-stranded DNA. Worldwide the current infective rate ranges between 40% and 100% [2]. Two studies in USA demonstrated that CMV positive rate in patients with acute severe colitis was 21–34%, in corticosteroid-refractory cases was 33–36%, and in active UC was 10% [3,4]. In Egypt, the CMV positive rate was 34.8% in corticosteroid-refractory IBD patients [2]. However, the prevalence of CMV in Chinese patients with IBD has not been reported in the literature till now.

CMV infection in IBD patients often makes clinical diagnosis and treatment complex. Maher et al. [2] have shown that IBD patients with CMV-positive were more likely to get fever, cervical lymphadenopathy, splenomegaly, leucopenia, thrombocytopenia, and pancolitis as compared to CMV-negative ones. Kandiel et al. [3] used antiviral drugs for the treatment of acute severe CMV-positive colitis, and achieved a remission rate of 67–100%. However, Lévêque et al. [5] found no relationship between CMV viral load and disease severity in patients with active IBD. Out of 7 CMV-positive patients treated with immunosuppressants and no antiviral therapy, remission was achieved in 5 patients. de Saussure et al. [6] treated 3 CMV-positive IBD cases with antiviral therapy, and only 1 patient got remission. Recently a study on cytomegalovirus infection in IBD undergoing treatment of anti-TNF-αantibody demonstrated that active CMV infection did not progress to CMV infection/disease following infliximab therapy, the response to infliximab therapy did not appear to be influenced by, or influence the course of, CMV infection/disease [7]. These studies aided in proving a link between CMV infection and refractory IBD. However, due to the small cohorts in some studies, further studies with larger cohorts need to be conducted in order to find a conclusive relationship between CMV and IBD.

Nested PCR of CMV-UL93 is considered to be a highly sensitive method for the detection of CMV [8]. Serum anti-CMV IgG and IgM are widely used in practice. The detection of IgG antibodies against CMV should be detected at the first visit, when the diagnosis of IBD is confirmed, in order to clarify whether the patient is at risk of displaying primary infection (IgG negative) or reactivation/reinfection (IgG positive). However, IgM antibodies to CMV are systematically detected in primary infection, reactivation or reinfection, it indicates CMV infection is in active stage. With primary infection, an early IgM antibody rise occurs and becomes detectable in the blood within the first week of infection. Anti-CMV IgM increases within 2 weeks of infection, and can remain positive for 3 months to 2 years. Its sensitivity and specificity for CMV infection could reach up to 100% and 98.6% respectively [9].

In this study, PCR detection of CMV DNA, and serological determinations of IgG and IgM were used to investigate the prevalence of CMV infection in IBD patients from central China. Moreover, risk factors for CMV infection in IBD patients were analyzed to find relationship between IBD and CMV infection.

## Methods

### Patients and healthy controls

From 2006 to 2011, 226 IBD patients (189 UC and 37 patients with CD) were recruited from Zhongnan Hospital of Wuhan University School of Medicine. The diagnosis of UC and CD was based on clinical, laboratory, imaging, endoscopic and histological examinations, and in accordance to the Chinese Medical Association diagnostic criteria for IBD [10]. Clinical disease activity of UC and CD was assessed by Truelove and Witts criteria [11] and Crohn’s disease activity index (CDAI) respectively [12].

Simultaneously, 290 sex and age matched healthy volunteers (controls) were recruited in Zhongnan Hospital Medical Center of Wuhan University, who attended this center for constitutional examination of health. All volunteers were unrelated to each other from Hubei province, and had no history of IBD, chronic infectious diseases, immune-mediated, ischemic and radiation-induced diseases. Subjects with history of use of corticosteroids and immunosuppressive agents, of drug abuse and unhealthy living were excluded from the study.

### Definitions

The patients and controls were classified as current smokers if they had smoked more than 1 cigarette per day within 6 months before recruited, and nonsmokers if they never or rarely smoked. As for definition of alcohol drinking before the onset of symptoms, 3 categories were included: frequent drinking, light drinking and former drinking. Frequent drinking was defined as alcohol drinking 3 days or more per week for continuous 6 months before recruited, excluded drink everyday to alcoholic intoxication. Light drinking was defined as alcohol beverages less than 3 days per week. Former drinking was defined as patients who had quit drinking for more than 6 months before recruited. They were all defined as drinking. Non-drinking was defined as never or rarely drinking. Mixed food was defined as the composition of diet include vegetable and meat for at least 6 months before recruited. The severity of UC was assessed by Truelove and Witts criteria [11]: Severe diarrhoea (six or more motions a day) with macroscopic blood in stools. Fever (mean evening temperature more than 37.5°C, or a temperature of 37.8°C, or more on at least two days out of four). Tachycardia (mean pulse rate more than 90 per minute). Anaemia (haemoglobin 75% or less-allowance made for recent transfusion). ESR much raised (more than 30 mm in one hour). Mild diarrhoea (four or less motions a day) with no more than small amounts of macroscopic blood in stools. No fever. No tachycardia. Anaemia not severe. ESR not raised above 30 mm. in one hour. Moderately-Intermediate between severe and mild [11]. The severity of CD was classified by Best CDAI: Index values between 150 and 220 are associated with mild disease; values between 220 and 450 are moderate disease; and values above 450 are seen with severe disease [12]. Drugs use was defined as taking related drugs for a period of at least 2 months to the time recruited.

### Ethics statement

The ethic committee of Zhongnan Hospital of Wuhan University approved the study. Consent was written by all subjects. Consent informed in current ethics statement was obtained from all participants involved in this study.

### Extraction of DNA

5 ml venous blood was taken in anticoagulated tubes with ethylenediamine tetraacetic acid (EDTA) from all subjects, followed by centrifugation. 2 ml of sera was taken and stored at −80 degrees for further anti-CMV IgG and IgM assay. Genomic DNA was extracted using proteinase K and phenol/chloroform method, which was then subsequently stored at −80 degrees.

### CMV-UL93 fragment detection

The CMV-UL93 fragment was retrieved from NCBI and BLAST, and was imported into Primer 5.0, according to the primer design principle. Lateral primers consisted of an upstream primer 5^′^-GGCAGCTATCGTGACTGGGA-3^′^, and a downstream primer 5^′^-GATCCGACC CATTGTCTAAA-3^′^. PCR conditions included 40 cycles of 95°C for 10 min, 95°C for 30 s, 57°C for 30 s, 72°C for 60 s, and then followed by 72°C for 10 min. Inner primers were an upstream primer 5^′^-TTAGCGCGT GACCTGTTACG-3^′^, and a downstream primer 5^′^-TCTAAATTGTTACGCAGTCCG-3^′^. PCR conditions included 40 cycles of 95°C for 10 min, 95°C for 30 s, 58°C for 30 s, 72°C for 60 s, and finally followed by 72°C for 10 min. Then electrophoresis using 2.5% agarose gel, of the medial segment was done to identify the products of PCR. Direct sequence for PCR products was done for detection of positive and negative PCR products. DNA-PCR+ was according to the result of electrophoresis of nested PCR, and ensured the result by DNA sequencing.

### Serum anti-CMV IgG and IgM detection

ELISA kit (DIESSE Diagnostica senese, Italy) was used to detect serum anti-CMV IgG and IgM in IBD patients and healthy controls. CMV IgG+ and CMV IgM+ were according to the kit: Positive defined as optical density (OD) ratio of sample to standard threshold value greater than 1.1, negative below 0.9.

### Histology and hematoxylin and eosin (H&E) staining

Colonoscopy and/or enteroscopy were conducted in anti-CMV IgM-positive IBD patients, and biopsies from pathological sites were taken. H&E staining for the detection of CMV was done.

### Statistical analysis

SPSS 13.0 software (SPSS for Windows version 13.0, Chicago, IL, USA) was used to conduct the statistical analysis. Measurement data are presented as mean ± standard deviation (SD), and numeration data are expressed as percentage and the number of cases. Independent samples were analyzed by Levene’s test. χ^2^(Chi-square) test with Yates continuity correction or Fisher’s exact test was performed to compare frequencies of risk factors between the IBD patients and healthy controls. Multiple logistic regression analysis was performed to evaluate multiple risk factors for IBD. Odds ratio (OR) and 95% confidence intervals (CI) were calculated. All calculated *P*-values were 2-sided and *P* < 0.05 was considered significant.

## Results

### Demographic and clinical profile

As shown in Table 1, Patients with inflammatory bowel disease and heathy controls were age- and sex-matched (P > 0.05), demographic and clinical profile were included in this table.

**Table 1:** Demographic and clinical profile of the inflammatory bowel disease patients and healthy controls

	** UC**	** CD**	** IBD**	**Healthy controls**	***P *value**
	** n = 189(%)**	** n = 37(%)**	** n = 226(%)**	** n = 290(%)**	
Mean age ± SD (yr)	40.81 ± 15.00	38.78 ± 14.14	40.66 ± 14.90	38.08 ± 14.63	0.888
Male/Female	109/80	23/14	132/94	170/120	0.961
Smoking	45 (23.81%)	9 (24.32%)	54 (23.89%)	101 (34.83%)	0.007*
Drinking	75 (39.68%)	12 (32.43%)	87 (38.50%)	133 (45.86%)	0.093
Diet composition					
vegetarian food	18 (9.52%)	6 (16.22%)	24 (10.62%)	19 (6.55%)	0.097
mixed food	171 (90.48%)	31 (83.78%)	202 (89.38%)	271 (93.45%)	
General state					
fever	32 (16.93%)	4 (10.81%)	36 (15.93%)		
hemoglobin (g/L)	114.10 ± 23.98	121.22 ± 25.34	115.71 ± 24.37		
albumin (g/L)	39.97 ± 5.88	38.12 ± 6.44	39.94 ± 6.04		
Disease extent					
distal colitis	140 (74.07%)				
pancolitis	49 (25.93%)				
ileal		8 (21.62%)			
colonic		14 (37.84%)			
ileocolonic		15 (44.11%)			
Disease severity					
mild/moderate	168	30			
severe	21 (11.1%)	7 (18.92%)			
Treatment					
ASA/SASP	149 (78.84%)	20 (54.05%)	169 (74.78%)		
corticosteroids	47 (24.87%)	8 (21.62%)	55 (24.34%)		
immunodepressant	5 (2.65%)	2 (5.41%)	7 (3.10%)		

*CMV*, cytomegalovirus; *UC*, ulcerative colitis; *CD*, Crohn’s disease; *ASA*, aminosalicylic acid; *SASP*, salicylazosulfapyridine.

*indicate significant difference.

### CMV-UL93 fragment, and CMV IgG, IgM detection

As shown in Table 2, prevalence of CMV-UL93 and anti-CMV IgG were significantly higher in IBD patients, than in healthy controls (84.07% vs 59.66%, *P <* 0.001; 76.11% vs 50.69%, *P <* 0.001), However, prevalence of anti-CMV IgM was no different with healthy controls (1.77% vs 0.34%, *P* = 0.235). For UC patients, CMV-UL93 and anti-CMV IgG were all significantly higher than in healthy controls (*P <* 0.001 and *P <* 0.001, respectively), While there was no difference between UC and healthy controls for anti-CMV IgM (*P =* 0.344). For CD patients, CMV-UL93 and anti-CMV IgG were higher than in controls (*P =* 0.027 and *P <* 0.001, respectively),while anti-CMV IgM was not increased as compared to healthy controls (*P* = 0.540).

**Table 2:** Prevalence of cytomegalovirus (CMV) in patients with inflammatory bowel disease and healthy controls

	** IBD**	** UC**	** CD**	**Healthy controls**
	** (n = 226)**	** (n = 189)**	** (n = 37)**	** (n = 290)**
CMV detection	n (%)	n (%)	n (%)	n (%)
DNA DNA+	90 (84.07)^a^	161 (85.19)^d^	29 (78.38)^g^	173 (59.66)
Serum CMV IgG+	172 (76.11)^b^	139 (73.54)^e^	33 (89.19)^h^	147 (50.69)
Serum CMV IgM+	4 (1.77)^c^	3 (1.59)^f^	1 (2.70)^i^	1 (0.34)

a Compare with healthy controls, OR = 3.569, 95% CI: 2.330-5.468, *P* < 0.001;

b Compare with healthy controls, OR = 3.099, 95% CI: 2.113-4.543, *P* < 0.001;

c Compare with healthy controls, OR = 5.207, 95% CI: 0.578-46.914, *P* = 0.235;

d Compare with healthy controls, OR = 3.889, 95% CI:2.443-6.190, *P* < 0.001;

e Compare with healthy controls, OR = 2.704, 95% CI:1.819-4.022, *P* < 0.001;

f Compare with healthy controls, OR = 4.661, 95% CI:0.481-45.148, *P =* 0.344;

g Compare with healthy controls, OR = 2.452, 95% CI:1.083-5.550, *P =* 0.027;

h Compare with healthy controls, OR = 8.026, 95% CI:2.772-23.232, *P* < 0.001;

i Compare with healthy controls, OR = 8.028, 95% CI:0.491-131.142, *P =* 0.540.

IBD, inflammatory bowel disease; CMV, cytomegalovirus; UC, ulcerative colitis; CD, Crohn’s disease.

However, in biopsies taken from the pathological sites of intestinal mucosa of anti-CMV IgM positive IBD patients, no inclusion bodies were detected in H&E stain.

### Univariate Analysis of Risk factors for CMV positive in patients with IBD

In UC patients, elevated CMV-DNA was mainly associated with the severity of disease activity (*P* = 0.048), use of ASA/SASP (aminosalicylic acid/salicylazosulfapyridine) and corticosteroids therapy (*P* = 0.041 and *P* = 0.035, respectively), while other factors, such as age, sex, smoking, alcohol consumption, type of diet, fever, anemia, albumin level, disease location, treatment with immunosuppressive agents had not shown significant association (*P > 0.05*). As for CD patients, CMV DNA positive was positively associated with use of 5-ASA/SASP (*P* = 0.014), as seen in Table 3.

**Table 3:** Univariate analysis of risk factors for CMV DNA positive in patients with inflammatory bowel disease

	**CMV DNA+/DNA-**					
	**UC**			**CD**		
	**(161/28)**			**(29/8)**		
Age	Number	*P* value	OR (95% CI)	Number	*P* value	OR (95% CI)
≤50 ys	117/22	0.514	0.725 (0.276 ~ 1.907)	18/6	0.685	0.545 (0.093 ~ 3.194)
>50 ys	44/6			11/2		
Sex						
male	95/14	0.373	1.439 (0.644 ~ 3.218)	19/4	0.445	1.900 (0.390 ~ 9.256)
female	66/14			10/4		
Smoking						
yes	40/5	0.423	1.521 (0.542 ~ 4.264)	7/2	1.000	0.840 (0.138 ~ 5.115)
no	121/23			25/6		
Drinking						
Yes	62/13	0.429	0.723 (0.322 ~ 1.621)	9/3	0.403	0.500 (0.100 ~ 2.510)
no	99/15			30/5		
Diet composition						
vegetarian food	15/3	1.000	0.856 (0.231 ~ 3.174)	3/3	0.101	0.192 (0.030 ~ 1.241)
mixed food	146/25			26/5		
Fever						
yes	30/2	0.221	2.977(0.670 ~ 13.235)	4/0	0.557	N/A
no	131/26			25/8		
Hemoglobin(G/L)						
≤70	8/1	1.000	1.412 (0.170 ~ 11.747)	0/0	-	N/A
>70	153/27			29/8		
Albumin(G/L)						
≤35	18/3	1.000	1.049 (0.288 ~ 3.826)	11/2	0.685	1.833 (0.313 ~ 10.735)
>35	143/25			18/6		
Disease severity						
mild and moderate	140/28	0.048*	N/A	24/6	0.631	1.600 (0.247 ~ 10.360)
severe	21/0			5/2		
Location						
distal colitis	123/17	0.080	2.094 (0.903 ~ 4.857)			
pancolitis	38/11					
ileal				7/1	0.734	N/A
Colonic				11/3		
ileocolon				11/4		
Treatment						
ASA/SASP	131/18	0.041*	2.426 (1.017 ~ 5.784)	19/1	0.014*	13.300 (1.429 ~ 123.790)
corticosteroids	45/2	0.035*	5.043 (1.149 ~ 22.128)	8/0	0.160	N/A
immunosuppressant	5/0	1.000	N/A	2/0	1.000	N/A

*CMV*, cytomegalovirus; *UC*, ulcerative colitis; *ASA*, aminosalicylic acid; *SASP*, salicylazosulfapyridine.

*indicate significant difference.

In UC patients, anti-CMV IgG level was associated with the use of 5-aminosalicylic acid (*P* < 0.001) as shown in Table 4. CD patients on vegetarian diet had much lower anti-CMV IgG positive rate, than those on non-vegeterian diet (*P* = 0.010). Other factors had no statistically significant impact on anti-CMV IgG positive rate (*P* > 0.05).

**Table 4:** Univariate analysis of risk factors for CMV IgG positive in patients with inflammatory bowel disease

	**CMV IgG+/IgG-**					
	**UC**			**CD**		
	**(139/50)**			**(33/4)**		
Age	Number	*P* value	OR (95% CI)	Number	*P* value	OR (95% CI)
≤50 ys	107/32	0.074	1.881 (0.934 ~ 3.786)	21/3	1.000	0.583 (0.054 ~ 6.251)
>50 ys	32/18			12/1		
Sex						
male	82/27	0.540	1.225 (0.639 ~ 2.349)	21/2	0.625	1.750 (0.218 ~ 14.069)
female	57/23			12/2		
Smoking						
yes	32/13	0.672	0.851 (0.404 ~ 1.793)	9/0	0.554	N/A
no	107/37			24/4		
Drinking						
Yes	53/22	0.467	0.784 (0.407 ~ 1.510)	10/2	0.582	0.435 (0.053 ~ 3.536)
no	86/28			23/2		
Diet composition						
vegetarian food	14/4	0.883	1.288 (0.403 ~ 4.115)	3/3	0.010*	0.033 (0.003 ~ 0.429)
mixed food	125/46			30/1		
Fever						
yes	26/6	0.278	1.687 (0.650 ~ 4.379)	4/0	1.000	N/A
no	113/44			29/4		
Hemoglobin (G/L)						
≤70	7/2	1.000	1.273 (0.255 ~ 6.341)	0/0	-	N/A
>70	132/48			33/4		
Albumin (G/L)						
≤35	16/5	0.771	1.171 (0.405 ~ 3.381)	11/2	0.602	0.500 (0.062 ~ 4.040)
>35	123/45			22/2		
Disease severity						
mild and moderate	120/48	0.109	0.263 (0.059 ~ 1.173)	26/4	0.570	N/A
severe	19/2			7/0		
Location						
distal colitis	108/32	0.058	1.960 (0.971 ~ 3.955)			
pancolitis	31/18					
ileal				7/1	0.219	N/A
Colonic				14/0		
ileocolon				12/3		
Treatment						
ASA/SASP	119/30	<0.001*	3.967 (1.897 ~ 8.296)	18/2	1.000	1.200 (0.150 ~ 9.570)
corticosteroids	38/9	0.190	1.714 (0.761 ~ 3.861)	8/0	0.557	N/A
immunosuppressant	5/0	0.328	N/A	2/0	1.000	N/A

*CMV*, cytomegalovirus; *UC*, ulcerative colitis; *ASA*, aminosalicylic acid; *SASP*, salicylazosulfapyridine.

*indicate significant difference.

The positive rate of anti-CMV IgM in UC patients, was associated with low albumin level (*P =* 0.031), severe UC (*P =* 0.031), use of corticosteroids (*P =* 0.015), and the use of immunosuppressive agents (*P* < 0.001), while other factors did not cause any statistically significant changes in anti-CMV IgM positive rate (*P* > 0.05). In CD patients, no statistically significant association with anti-CMV IgM was found, as shown in Table 5.

**Table 5:** Univariate analysis of risk factors for CMV IgM positive in patients with inflammatory bowel disease

	**CMV IgM+/IgM-**					
	**UC**			**CD**		
	**(3/186)**			**(1/36)**		
Age	Number	*P* value	OR (95% CI)	Number	*P* value	OR (95% CI)
≤50 ys	2/137	1.000	0.715 (0.063 ~ 8.065)	1/23	1.000	N/A
>50 ys	1/49			0/13		
Sex						
male	2/107	1.000	1.477 (0.132 ~ 16.573)	1/22	1.000	N/A
female	1/79			0/14		
Smoking						
yes	1/44	1.000	1.614 (0.143 ~ 18.222)	1/8	0.243	N/A
no	2/142			0/28		
Drinking						
yes	1/74	1.000	0.757 (0.067 ~ 8.496)	0/12	1.000	N/A
no	2/112			1/24		
Diet composition						
vegetarian food	0/18	1.000	N/A	0/6	1.000	N/A
mixed food	3/168			1/30		
Fever						
yes	1/31	1.000	2.500 (0.220 ~ 28.432)	0/4	1.000	N/A
no	2/155			1/32		
Hemoglobin (G/L)						
≤70	1/8	0.329	11.125 (0.911 ~ 135.909)	0/0	-	N/A
>70	2/178			1/36		
Albumin (G/L)						
≤35	2/19	0.031*	17.579 (1.522 ~ 203.083)	1/12	0.351	N/A
>35	1/167			0/24		
Disease severity						
mild and moderate	1/167	0.031*	0.057 (0.005 ~ 0.657)	1/29	1.000	N/A
severe	2/19			0/7		
Location						
distal colitis	1/139	0.337	0.169 (0.015 ~ 1.907)			
pancolitis	2/47					
ileal				1/7	0.155	N/A
Colonic				0/14		
ileocolon				0/15		
Treatment						
ASA/SASP	3/146	1.000	N/A	1/19	1.000	N/A
corticosteroids	3/44	0.015*	N/A	0/8	1.000	N/A
immunosuppressant	3/2	<0.001*	N/A	0/2	1.000	N/A

*CMV*, cytomegalovirus; *UC*, ulcerative colitis; *CD*, Crohn’s disease; *OR*, Odds ratio; *CI*, confidence interval; *ASA*, aminosalicylic acid; *SASP*, salicylazosulfapyridine.

*indicate significant difference.

### Multivariate analysis by logistic regression for CMV positive in IBD

As shown in Table 6. In multivariate analysis of risk factors in UC, pancolitis was significantly associated with CMV DNA elevated (*P* = 0.001). Severe UC and pancolitis seemed to be related with IgG positive (*P* = 0.021 and *P* = 0.017, respectively). For CD, there was just single factor associated with CMV positive in each group, multivariate analysis was unnecessary.

**Table 6:** Multivariate analysis by logistic regression for CMV positive in ulcerative colitis

**Index**	** OR (95% CI)**	***P***
**CMV DNA**		
Location	0.227 (0.093 ~ 0.553)	0.001*
Disease severity	0.000 (0.000~)	0.998
Fever	1.704 (0.357 ~ 8.138)	0.504
Treatment of corticosteroids	0.574 (0.228 ~ 1.449)	0.240
Treatment of ASA/SASP	1.761 (0.661 ~ 4.691)	0.258
**CMV IgG**		
Age	0.893 (0.398 ~ 2.005)	0.784
Location	2.482 (1.145 ~ 5.380)	0.021*
Disease severity	0.078 (0.009 ~ 0.635)	0.017*
Treatment of ASA/SASP	0.749 (0.342 ~ 1.639)	0.470
**CMV IgM**		
Alb	0.000 (0.000~)	0.997
Disease severity	0.840 (0.000~)	1.000
Treatment of corticosteroids	1.038 (0.000~)	1.000
Treatment of immunodepressant	0.997 (0.000~)	0.997

*CMV*, cytomegalovirus; *OR*, Odds ratio; *CI*, confidence interval; Alb, albumin; *ASA*, aminosalicylic acid; *SASP*, salicylazosulfapyridine.

*indicate significant difference.

## Discussion

CMV is an opportunistic pathogenic microorganism. *In vivo* it can proliferate in epitheliums, white blood cells and sperm cells, and it is prone to cause latent infection of salivary gland, mammary gland, kidney and white blood cells. In IBD patients, immunosuppressive therapy, impaired absorption of nutrients, dysfunction of the immune system, render them susceptible to CMV infection [13], which is consistent with the high infective rate of CMV in immunosuppressed individuals, such as acquired immunodeficiency syndrome (AIDS), transplant recipients, cancer, chemotherapy, but rare in immunocompetent individuals.

The detection methods of CMV infection in IBD patients included DNA detection, serological tests (serum anti-CMV IgM, IgG), histopathology (inclusion bodies detection) in this study. Body fluids or tissue sample was feasible to CMV culture, but it was time-consuming and also had low sensitivity, which limited clinical application [14]. The detection of CMV-DNA was considered as the most sensitive method [15], but it was associated with false positive results, thereby decreasing its specificity. Increase in serum anti-CMV IgM level occurred in recent CMV infection and it had high sensitivity and specificity [9], while anti-CMV IgG indicated past CMV infection. Sensitivity of H&E was 10% to 87% [3], CMV inclusion bodies could be found in biopsy specimens from colon with inflamed and ulcerated mucosa [16]. We used CMV-DNA specific fragment UL93, anti-CMV IgG and IgM to detect CMV infection in IBD patients and healthy controls. Positive rates in IBD patients were 84.07%, 76.11%, 1.77% for CMV DNA, anti-CMV IgG and IgM respectively, and 59.66%, 50.69%, 0.34% for healthy controls. CMV-UL93 and anti-CMV IgG were significantly higher in IBD patients as compared to controls, thereby indicating the association between IBD and CMV.

The positive rate of CMV UL-93 or anti-CMV IgG in the healthy controls was about 50–60%, whereas in IBD patients it was around 70–80%. A research conducted in India showed the CMV DNA positive rate was just 12.70% in IBD patients [17], which was remarkably lower than in our country. The small number of subjects enrolled (63 IBD patients) could account for the low positive rate in the Indian study. Another research done in France showed 60% positive rate in IBD patients [18], which was consistent with our research. In developed countries, the anti-CMV IgG positive rate was found to be above 70% [19].

Anti-CMV IgM positive rate is only 1.77% in our research, which was lower as compared to an Indian study, including IBD patients with both active disease and in remission [17], where the rate was 9.52%. Anti-CMV IgM positive rate in healthy controls (0.34%) was no different with IBD patients (*P* = 0.235), which was related with small amount of people included.

Presence of anti-CMV IgM indicated recent infection of CMV. However in biopsies taken from the pathological sites of intestinal mucosa of anti-CMV IgM positive IBD patients, no inclusion bodies was detected. This was probably due to the low sensitivity of histological examination. The site from where the biopsy was taken, and the amount of tissue retrieved, may also influence the sensitivity of finding inclusion bodies. Next stage we should recruit enough biopsies and screen CMV by immunohistochemistry.

In a model of multivariate analysis adjusting for multiple factors for UC. Disease location seemed to be significantly associate with CMV infection or re-activation, this may be associated with the theory that CMV was prone to proliferate in granulation tissue [20]. Pancolitis involved with larger areas of ulcerative mucosa, which promote proliferation of CMV. Some researches [21] found that CMV was readily discovered in granulation tissue and tissue from deep ulcers, which suggested that CMV could penetrate inflamed mucosa via mononuclear cells, and then proliferate in the mucosa. A recent study [22] showed that the murine CMV (MCMV) could not induce acute colitis, but the latent MCMV infection could increase the severity of the dextran sulfate sodium (DSS) induced colitis. Moreover, acute MCMV infection could significantly increase the serum and intestinal natural killer cells, interleukin (IL)-6, TNF-α, IFN-γ, indicating that CMV infection can modulate mucosal immunity, thereby increasing susceptibility to inflammation. CMV infection can also activate oncogenes, kinases, transcription factors inducing tumorigenesis, which may be one of the reasons of the higher incidence of colorectal cancer in IBD patients [23]. CMV played a role in the initiation and progression of inflammation in IBD. The treatment of 5-ASAs, corticosteroids and immunosupressents were no longer significant associated with CMV infection in multivariate analysis.

Currently there is no absolute indication for antiviral therapy in CMV-positive IBD patients. However Eddleston recommends antiviral therapy in immunocompetent [24]. Pfau [21] found out that ganciclovir could reduce mortality rate and surgical intervention rate, while de Saussure P [6] showed that antiviral therapy had no effect on the disease course.

In summary, as compared to healthy individuals, IBD patients have a predisposition to CMV infection. No risk factor was found to be significantly correlated with CMV infection in risk factors analysis.

## Abbreviations

CMV: Cytomegalovirus; IBD: Inflammatory bowel disease; UC: Ulcerative colitis; CD: Crohn’s disease; PCR: Polymerase chain reactio; OR: Odds ratio; CI: Confidence interval; Alb: Albumin; H&E: Hematoxylin and eosin; CDAI: Crohn’s disease activity index; SD: Standard deviation; ASA: Aminosalicylic acid; SASP: Salicylazosulfapyridine.

## Competing interests

The authors declare that they have no conflict of interests.

## Authors’ contributions

FY, JZ, YL and BX conceived the study, participated in its design and coordination, and managed the preparation of the manuscript. CW and SH carried out the nested PCR, FY and LS carried out ELISAs, All authors participated in the recruitment of specimens and clinical data of patients and healthy controls. RL and FY participated in the manuscript writing. BX and FY performed the statistical analysis. WW participated in the manuscript revision. All authors read and approved the final manuscript.
